# Structural insights into the octamerization of glycerol dehydrogenase

**DOI:** 10.1371/journal.pone.0300541

**Published:** 2024-03-14

**Authors:** Taein Park, Jung Youn Kang, Minwoo Jin, Jihyeong Yang, Hyunwoo Kim, Chaemin Noh, Che-Hun Jung, Soo Hyun Eom

**Affiliations:** 1 Department of Chemistry, Gwangju Institute of Science and Technology (GIST), Gwangju, Republic of Korea; 2 School of Life Sciences, Gwangju Institute of Science and Technology (GIST), Gwangju, Republic of Korea; 3 Department of Molecular Medicine, Chonnam National University, Gwangju, Republic of Korea; 4 Department of Chemistry, Chonnam National University, Gwangju, Republic of Korea; University of Minnesota, The Hormel Institute, UNITED STATES

## Abstract

Glycerol dehydrogenase (GDH) catalyzes glycerol oxidation to dihydroxyacetone in a NAD^+^-dependent manner. As an initiator of the oxidative pathway of glycerol metabolism, a variety of functional and structural studies of GDH have been conducted previously. Structural studies revealed intriguing features of GDH, like the flexible β-hairpin and its significance. Another commonly reported structural feature is the enzyme’s octameric oligomerization, though its structural details and functional significance remained unclear. Here, with a newly reported GDH structure, complexed with both NAD^+^ and glycerol, we analyzed the octamerization of GDH. Structural analyses revealed that octamerization reduces the structural dynamics of the N-domain, which contributes to more consistently maintaining a distance required for catalysis between the cofactor and substrate. This suggests that octamerization may play a key role in increasing the likelihood of the enzyme reaction by maintaining the ligands in an appropriate configuration for catalysis. These findings expand our understanding of the structure of GDH and its relation to the enzyme’s activity.

## Introduction

For various microbes, including *Escherichia coli* (*E*. *coli*), glycerol serves as a carbon source to produce energy, especially under anaerobic conditions [1–4]. Glycerol is metabolized via coupled oxidative and reductive pathways, with glycerol dehydrogenase (GDH; EC 1.1.1.6) serving as the initiator enzyme for the oxidative pathway [3, 5, 6]. GDH catalyzes the oxidation of glycerol to dihydroxyacetone (DHA) with concomitant reduction of NAD^+^ to NADH. As a member of the family III metal-containing polyol dehydrogenases, GDH requires not only NAD^+^, but also a metal ion, typically Zn^2+^, for its activity [6, 7].

Numerous studies have been conducted investigating the biochemical and functional characteristics of GDH from various species. Kinetic studies examining the initial velocity and product inhibition confirmed that GDH follows an ordered Bi-Bi kinetic mechanism, where NAD^+^ binding to the apo enzyme is the first step and NADH release is the last [8–10]. The glycerol oxidation catalyzed by GDH consists of a hydride shift from C_2_ of glycerol to the C_4_^N^ atom of NAD^+^, with the typical distance between the donor and acceptor for the hydride shift being approximately 2.6 Å [11–13]. In addition, GDH is reported to have relatively high thermal stability, with a *T*_m_ of 60 to 80°C across various species, including non-thermophilic microorganisms like *E*. *coli* [14–16]. GDH activity is also reportedly pH-dependent, with an optimum pH of approximately 10 for the forward reaction [14–16].

The structure of GDH features two well-separated domains, the N- and C-domains; between them is a central cleft where the active site is located [17–23]. The N-domain includes six parallel β-sheets and ɑ-helices in a Rossmann fold arrangement, while the C-domain includes several ɑ-helices forming bundles [17, 19]. An intriguing structural feature of GDH is the β-hairpin (β7–8) located between the two domains, which exhibits marked dynamic conformational variation depending on the presence of NAD^+^ [23]. The flexibility of this β-hairpin was revealed to contribute to the enzyme’s efficiency. Another noteworthy characteristic is the homo-octameric configuration observed for all reported GDH structures, with the exception of the enzyme from the evolutionarily distant *Clostridium acetobutylicum* (*C*. *acetobutylicum*) [17–23]. This structural attribute has also been observed in solution-based assays with various species, including *E*. *coli* [8, 15–17, 24]. However, the significance of the octamerization of GDH to its enzyme activity has yet to be investigated.

In the present study, we analyzed a newly reported crystal structure of *E*. *coli* GDH complexed with both NAD^+^ and glycerol (GDH^NAD∙Gly^) to elucidate the octamerization of GDH. Molecular dynamics (MD) simulations revealed that within the GDH octamer, the structural dynamics of the N-domain are reduced compared to the monomer, which allows for a closer and more consistent distance between the cofactor and substrate for the catalytic reaction. These findings suggest a potential contribution of GDH’s octamerization to its enzyme activity.

## Materials and methods

### Cloning and expression of the recombinant protein

The gene encoding full-length *E*. *coli* GDH (EC 1.1.1.6) was amplified from the genomic DNA of *E*. *coli* K12 MG1655 using polymerase chain reaction. The amplified DNA and the pET21a(+) plasmid vector, featuring an N-terminal six-histidine (His_6_) tag, underwent *Nde*I and *Xho*I restriction enzyme treatments, and were ligated together using DNA ligase. *E*. *coli* BL21 (DE3) cells were then transformed with the resulting recombinant plasmid for protein expression. The cells were cultured in Luria-Bertani (LB) broth containing 50 μg/mL ampicillin at 37°C until they reached an optical density at 600 nm of 0.6. Overexpression of the recombinant protein was induced by adding isopropyl β-D-1-thiogalactopyranoside to a final concentration of 0.2 mM, after which the cells were incubated at 37°C for an additional 4 h. The cells were then harvested by centrifugation at 5,000 × g for 15 min at 4°C.

### Purification of the recombinant protein

The harvested cells expressing the recombinant *E*. *coli* GDH were resuspended in a buffer containing 50 mM HEPES-NaOH (pH 7.5), 1 mM EDTA, and 1 mM phenylmethylsulfonyl fluoride. The cells were then disrupted by sonication, after which the cell lysate was centrifugated at 13,000 × g for 30 min at 4°C. The soluble fraction was loaded onto a gravity-flow open column packed with Ni-IDA agarose resin, which had been pre-equilibrated with a buffer containing 50 mM HEPES-NaOH (pH 7.5) and 10 mM imidazole. After washing with another HEPES buffer where the imidazole concentration was increased to 50 mM, the target protein was eluted with 300 mM imidazole in the same buffer. The eluate was further purified through size-exclusion chromatography using a HiLoad 16/600 Superdex 200 prep grade column with a 50 mM HEPES-NaOH buffer (pH 7.5). The GDH-containing fractions were collected, concentrated to 12 mg/mL, and stored at -80°C.

### Crystallization

Prior to crystallization, ZnCl_2_ and NAD^+^ were added to the purified GDH sample to final concentrations of 1 mM and 2 mM, respectively. The GDH^NAD∙Gly^ crystal was prepared using methods similar to those previously reported [23]. The refined GDH^NAD∙Gly^ crystal was obtained using the hanging-drop vapor diffusion method with a 1:1 mixture of protein solution and well solution containing 100 mM sodium phosphate dibasic and 100 mM citric acid (pH 4.2 adjusted with NaOH), 200 mM NaCl, and 10% (*w*/*v*) PEG 3000 at 20°C. The crystal was soaked for 1 h in the well solution supplemented with 25% PEG 3000 and 5% (*v*/*v*) glycerol for both cryo-protection and ternary complex formation, then immediately flash-frozen in liquid nitrogen for data collection.

### Data collection and structure determination

The diffraction data for the GDH^NAD∙Gly^ crystal were collected using a single wavelength of 0.9795 Å and the Pilatus3 6M detector at beamline 11C at the Pohang Accelerator Laboratory, South Korea. The collected diffraction data were processed using DIALS [25]. The crystal diffracted to a resolution of 2.0 Å and belonged to the *I*422 space group. Using PHASER in the CCP4 suite [26], the structure was determined using the molecular replacement method with a single chain from the reported *E*. *coli* GDH structure (PDB: 8GOB) serving as the template. The structure was then refined using REFMAC5 in the CCP4 suite [27] and manually using COOT [28] to a *R*_work_/*R*_free_ of 18.9%/22.0%. The structural model was validated using the PDB validation web server. The coordinate and structural factors were deposited in the PDB RCSB with an accession code 8X6M. The statistics for the data collection and refinement are presented in Table 1. All structural figures were generated using PyMOL version 2.5.0 (Schrödinger LLC, New York, NY, USA).

**Table 1:** Data collection and refinement statistics.

	GDH^NAD∙Gly^
Data collection	
X-ray source	PAL 11C
Space group	*I*422
Wavelength (Å)	0.9795
Unit-cell parameters	
*a*, *b*, *c* (Å)	131.9, 131.9, 265.3
*ɑ*, *β*, *γ* (°)	90.0, 90.0, 90.0
Resolution range (Å)	118.1–2.0 (2.03–2.00)
No. of unique reflections	78784 (3865)
*R*_sym_	0.107 (0.962)
*R*_pim_	0.030 (0.268)
CC_1/2_	0.999 (0.940)
<*I*/σ(*I*)>	19.0 (1.9)
Completeness (%)	99.9 (99.4)
Redundancy	26.1 (26.5)
**Refinement**	
Resolution range (Å)	50.0–2.0
*R*_work_ (%) / *R*_free_ (%)	18.9 / 22.0
No. of atoms	
Protein	5446
Ions (Zn^2+^)	2
Ligands (NAD^+^ and glycerol)	100
Water	429
Overall averaged *B*-factor (Å^2^)	
Protein (Mol-A / Mol-B)	37 / 39
Zn^2+^ (Mol-A / Mol-B)	81 / 120
NAD^+^ (Mol-A / Mol-B)	39 / 110
Glycerol (active site / secondary)	79 / 74
RMSD	
Bond lengths (Å)	0.008
Bond angles (°)	1.572
Ramachandran plot (%)	
Favored / allowed / disallowed	98 / 2 / 0

### Enzyme assay

The concentrations of NADH produced at the optimum or lower pH were calculated by measuring its absorbance at a wavelength of 340 nm at 20°C using a UV-Vis spectrophotometer. The purified GDH sample used for the crystallization was also used for this assay, and the reaction mixtures were prepared taking into consideration the crystallization conditions of the GDH^NAD⋅Gly^ crystal. Mixtures containing 0.4 μM GDH, 600 μM NAD^+^, 100 mM glycerol, 200 mM NaCl, and 25% PEG 3000 in 100 mM sodium phosphate/citrate-NaOH buffer (pH 4.2) or 100 mM CHES-NaOH buffer (pH 10.0) were incubated at 20°C for 1 h, after which the NADH concentration in each mixture was assessed. One unit of enzyme activity was defined as the amount of enzyme that catalyzed the conversion of 1 μmol of substrate per minute at 20°C, and the specific activity was expressed as units/mg of enzyme.

### Molecular dynamics simulation and analysis

For simulations, models of GDH in three ligand-bound states were prepared using methods similar to those previously reported [23], but with molecules forming an octamer according to crystallographic symmetry. The simulations were carried out using the GROMACS version 2021.3 software package [29], with the Amber99SB-ILDN force field [30] for the protein and the ff14SB force field [31] for the ligands. Molecular topologies of the ligands were generated using ACPYPE [32]. TIP3P water molecules [33] were used to solvate the system in a cubic box. To neutralize the system containing the charged protein, Na^+^ or Cl^−^ were added as counter ions. Energies of the systems were then minimized using the steepest descent method. The bond lengths were constrained using LINCS [34], and long-range electrostatic interactions were calculated using the Particle Mesh Ewald method [35, 36]. The systems were equilibrated for 100 ps under the NVT condition at 300 K [37], then for 100 ps under the NPT condition at 300 K and 1 bar [38–41]. MD productions were conducted for 20 ns under the same NPT condition with no restraints. The time steps were 2 fs, and snapshots were taken every 10 ps. To analyze the results, the snapshots were clustered, providing 20 representative structures for each simulation. The structures for each were then aligned based on the backbones, after which root-mean-square fluctuations (RMSFs) for the C_α_ atoms per residue were calculated and plotted.

## Results

### Structure of the GDH ternary complex with NAD^+^ and glycerol

Our previous structural study of GDH complexed with NAD^+^ and an inhibitor, tris(hydroxymethyl)aminomethane (Tris), provided insight into the GDH ternary complex [23]. However, due to the divergent chemical properties of Tris compared to glycerol, it remained uncertain whether the ternary complex elucidated was the same as that with the actual substrate bound. To address this, we endeavored to determine the crystal structure of GDH complexed with its cofactor NAD^+^ and substrate glycerol (GDH^NAD∙Gly^). Full-length *E*. *coli* GDH (1–367) was crystallized in the presence of NAD^+^ and then soaked in glycerol-containing solution for 1 h before flash-freezing in liquid nitrogen for data collection.

**Fig 1:**
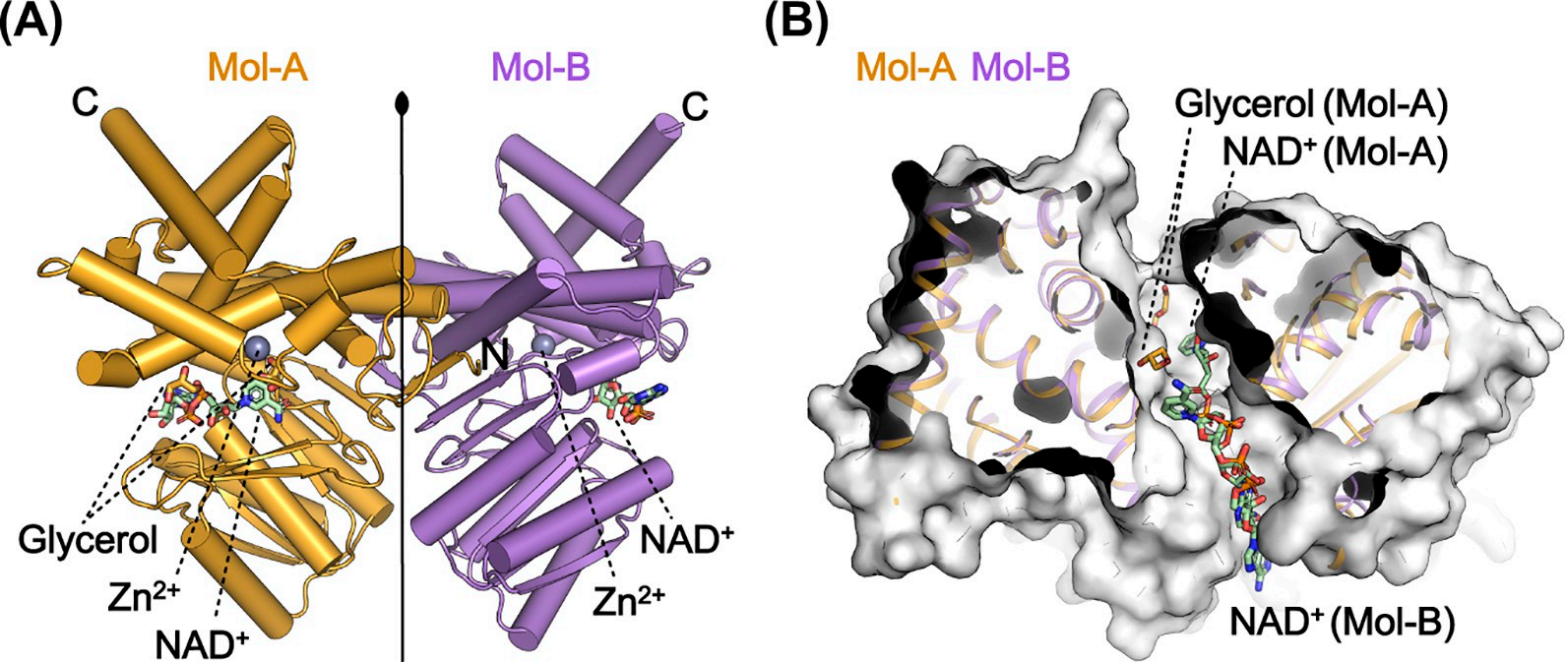
Overall structure of GDH^NAD∙Gly^. (A) Overall structure of two protomers in the GDH^NAD∙Gly^ structure. Mol-A and Mol-B are colored orange and purple, respectively. NAD^+^ and glycerol are shown in stick, and Zn^2+^ is shown as a grey sphere. (B) Structural superposition of the two protomers, showing the ligands on the central cleft. Mol-A and Mol-B are colored as in (A), and the ligands of each protomer are presented in stick.

**Fig 2:**
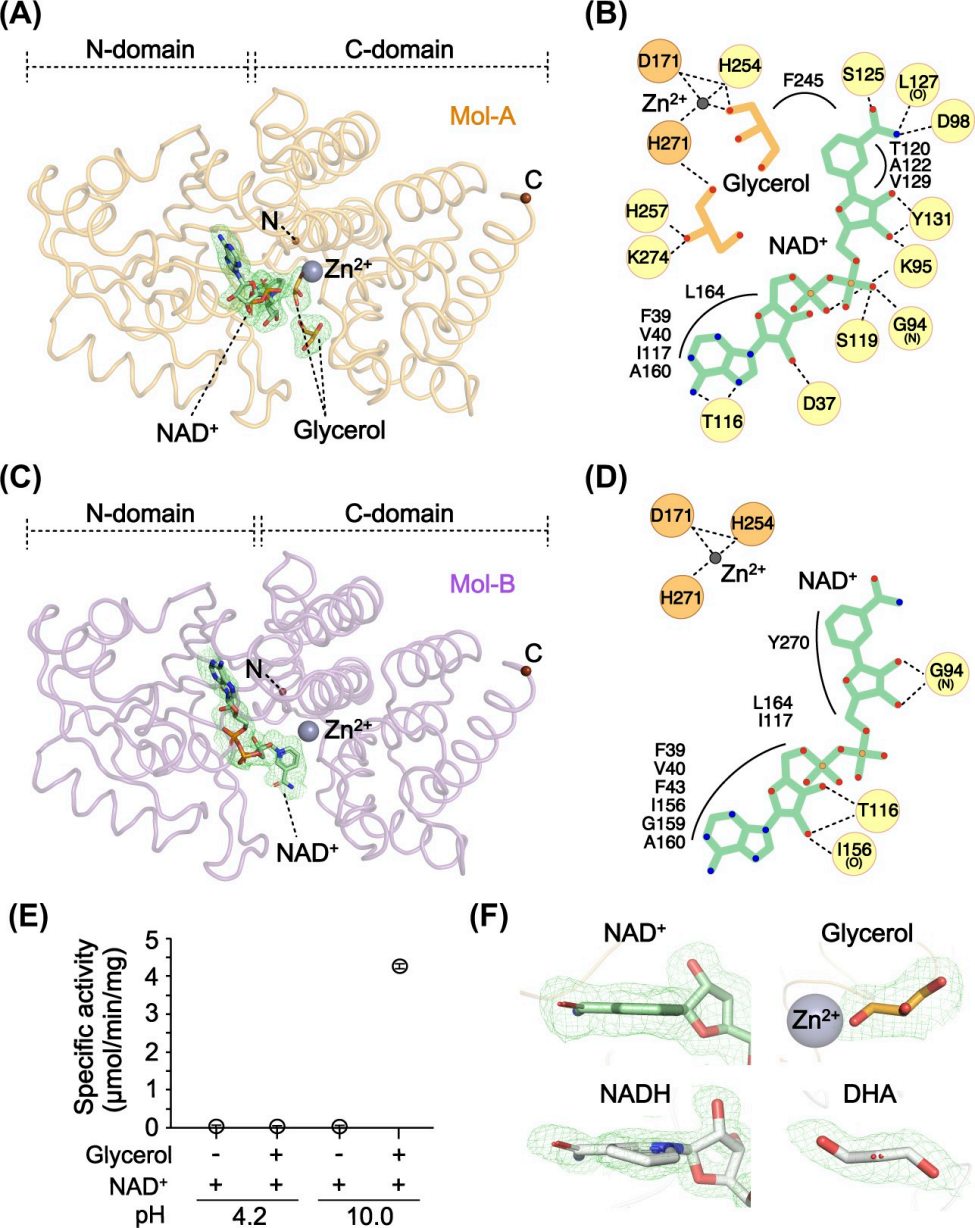
Ligand binding of two protomers of GDH^NAD∙Gly^. (A) Mol-A of the GDH^NAD∙Gly^ structure with simulated annealing omit *F*_o_-*F*_c_ maps for each ligand contoured at 3.0 σ. The N- and C- terminals are marked as brown spheres. NAD^+^ and glycerol are shown in stick, and Zn^2+^ is shown as a grey sphere. (B) Schematic representation of the interactions between the ligands and their surroundings in Mol-A within the GDH^NAD∙Gly^ structure. Hydrogen bonds are shown as dashed lines, and the participating residues are circled in yellow. Residues participating in hydrophobic interactions are shown with curved lines. (C) Mol-B in the GDH^NAD∙Gly^ structure with a simulated annealing omit *F*_o_-*F*_c_ map for NAD^+^. The two terminals and the ligands are shown as in (A). (D) Schematic representation of the interactions between NAD^+^ and its surroundings in Mol-B within the GDH^NAD∙Gly^ structure shown as in (B). (E) Specific activities of *E*. *coli* GDH under four conditions; in the presence of NAD^+^, enzyme activity was measured with or without glycerol at pH 4.2 or 10.0. Data are shown as the mean ± 95% confidence intervals (95CI) for triplicate experiments. (F) Conformational comparisons between NAD^+^ (GDH^NAD∙Gly^) and NADH (from a horse liver alcohol dehydrogenase structure; PDB: 8G41) or between glycerol (GDH^NAD∙Gly^) and DHA (from a DHA kinase structure; PDB: 3PNQ) shown with their simulated annealing omit *F*_o_-*F*_c_ maps contoured at 3.0 σ or their 2*F*_o_—*F*_c_ maps contoured at 1.0 σ (green meshes).

Within the asymmetric unit of the GDH^NAD∙Gly^ structure, there were two molecules (Mol-A and Mol-B) related by a 2-fold non-crystallographic symmetry (Fig 1A). Interestingly, the ligand-bound states of each protomer was distinct (Fig 1B). Mol-A formed a ternary complex with both ligands bound at the active site (Fig 2A). The binding mode of NAD^+^ was consistent with previously reported observations, and glycerol coordinated with Zn^2+^ through its C_1_-OH oxygen in a non-productive manner or was positioned at a secondary binding site (Fig 2B). By contrast, Mol-B had only NAD^+^ bound without glycerol, and the binding was at an alternative site along the central cleft (Figs 1B, 2C and 2D). It appears that one of the intermediate states in NAD^+^ access or release was captured.

All the ligands were fitted as their respective reactant forms, considering the low pH of the crystallization conditions and the shapes of their electron density maps. The pH under the crystallization conditions was 4.2, significantly lower than the optimum pH of approximately 10.0 for the forward enzymatic reaction of GDH. It has been reported that glycerol oxidation rarely occurs at such a low pH [16, 42]. Consistent with that report, when we compared the specific activities of GDH at pH 4.2 and 10.0, we found that at pH 4.2 the enzyme exhibited only 0.4% of the activity it showed at pH 10.0 (Fig 2E). Furthermore, the shapes of the electron density maps for each ligand appeared more consistent with their respective reactant forms, with no puckering of the benzene ring of the NAD^+^ nicotinamide moiety and with glycerol in a non-flat configuration (Fig 2F). Collectively, this newly reported GDH^NAD∙Gly^ structure shows that the ligand-bound states of each protomer comprising the GDH homo-oligomer are independently adopted.

### Octameric oligomerization of GDH

**Fig 3:**
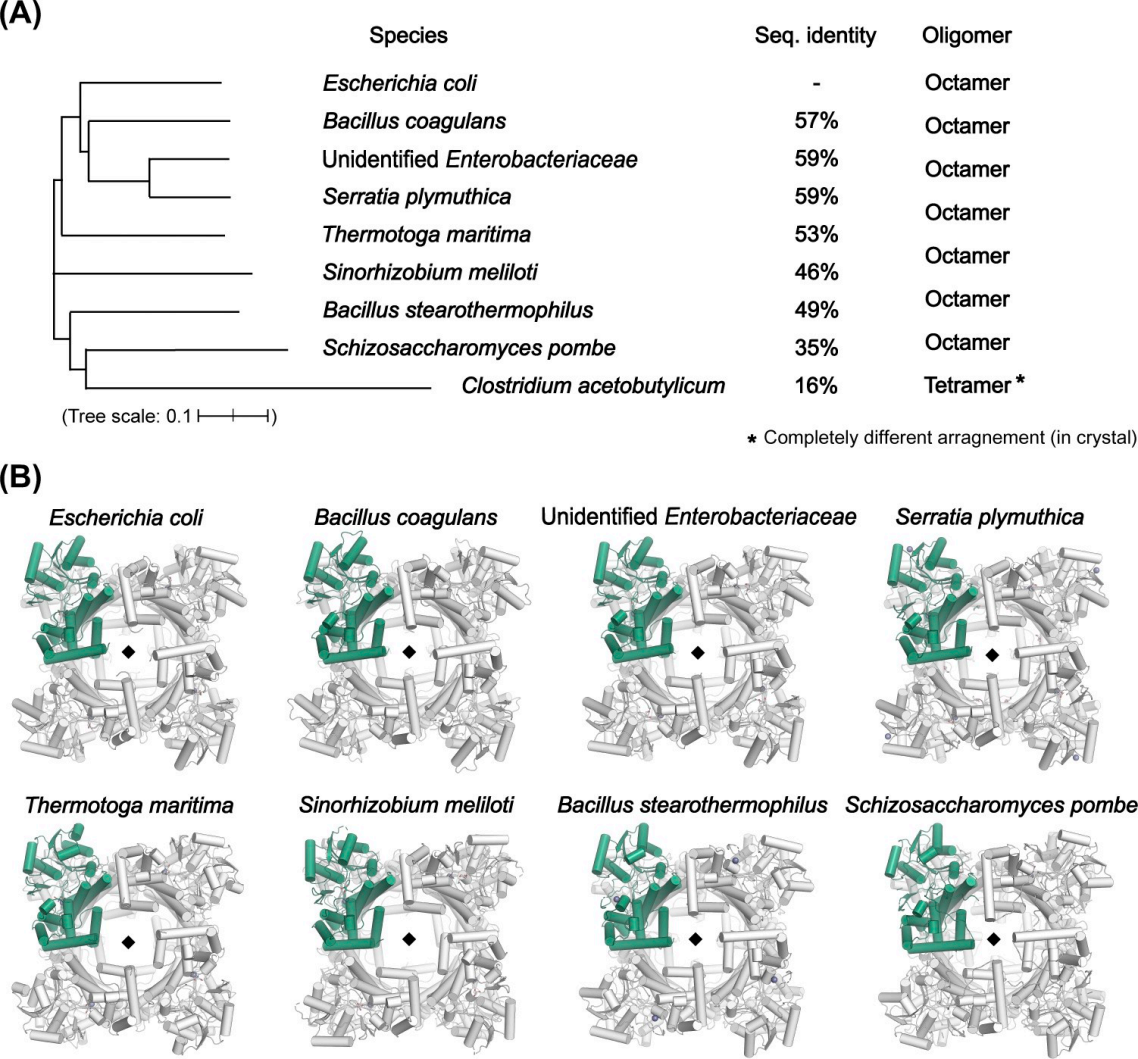
Oligomeric states of GDH crystal structures from various species. (A) Phylogenetic analysis of the *E*. *coli* GDH structure and reported GDH structures from eight other species. The aligned sequences were plotted using Interactive Tree of Life (iTOL). Sequence identities were compared with *E*. *coli* GDH, and oligomeric states of each GDH crystal structure are presented (PDB: 5ZXL, 6CSJ, 5XN8, 4MCA, 1KQ3, 3UHJ, 1JQA, and 1TA9, in order). (B) GDH octamers from the eight reported crystal structures as in (A). The unit protomer is colored green. The 4-fold rotational axes in the structures are presented as black diamonds.

**Fig 4:**
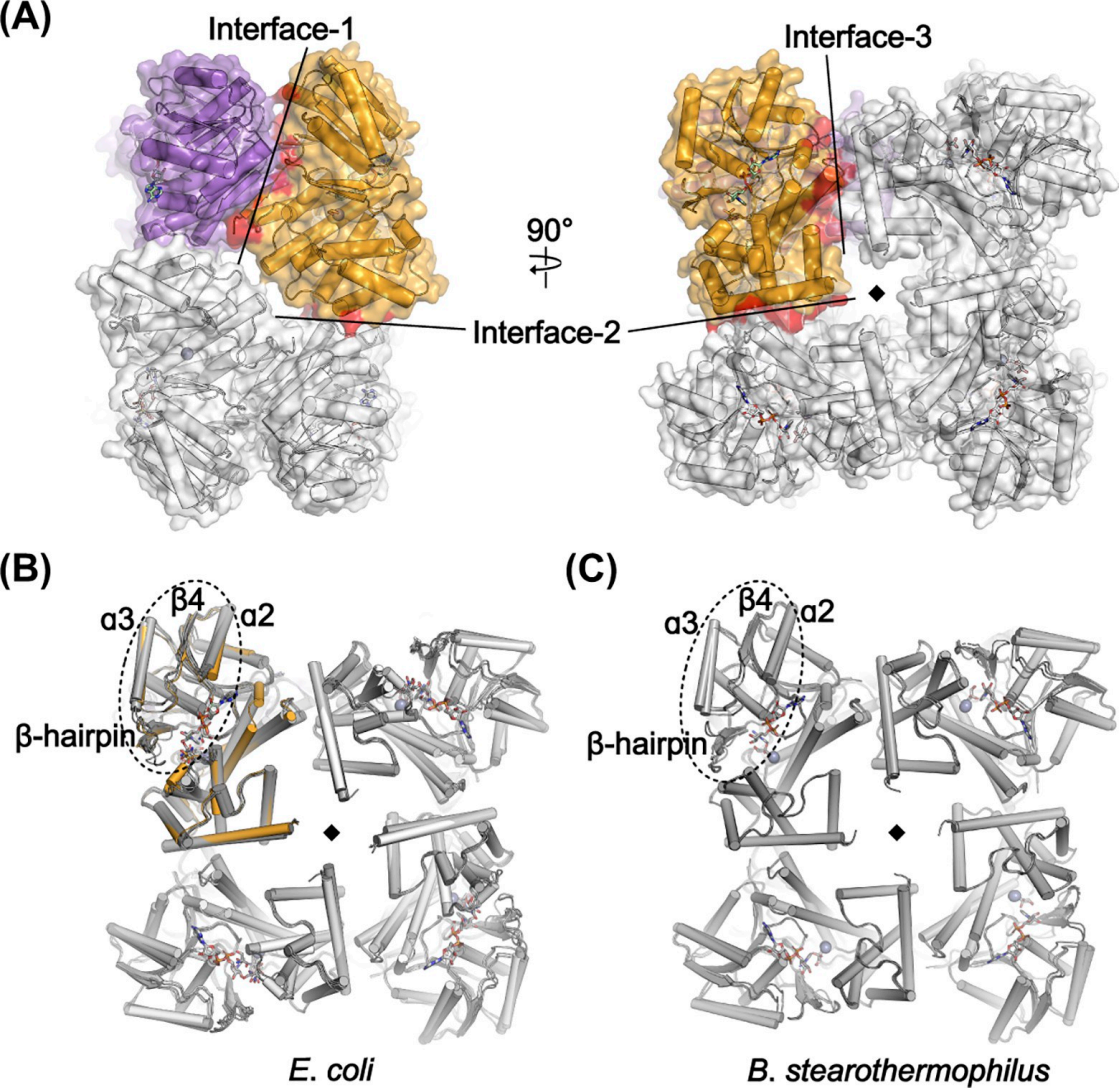
Octameric interfaces of GDH^NAD∙Gly^. (A) Octameric structure of GDH^NAD∙Gly^. Mol-A and Mol-B are colored orange and purple, respectively. Three octameric interfaces per protomer are shown as red surfaces in Mol-A. (B and C) Structural superposition of four *E*. *coli* GDH structures, including the GDH^NAD∙Gly^ structure (B), and three *B*. *stearothermophilus* GDH structures (C). Mol-A of the GDH^NAD∙Gly^ structure in (B) is colored orange. Regions showing major structural differences are indicated by dashed circles. The 4-fold rotational axes in the structures are presented as black diamonds.

With the exception of the enzyme from the evolutionarily distant *C*. *acetobutylicum*, all previously reported GDH structures exhibited octameric configurations related to their crystallographic symmetries (Fig 3) [17–22]. Despite the two protomers adopting distinct ligand-bound states, the present GDH^NAD∙Gly^ structure also adopted an octameric structure through crystallographic 4-fold symmetry. Our size-exclusion chromatography experiment, conducted using a HiLoad 16/600 Superdex 200 prep grade column with 50 mM HEPES-NaOH buffer (pH 7.5), also revealed a protein peak corresponding to GDH at a size consistent with its octamer (S1 Fig). This octamerization entailed formation of three interfaces per protomer: Interface-1 in the N-domain and Interfaces-2 and -3 in the C-domain (Fig 4A). These octameric interfaces were consistent across all GDH octameric structures, with an average root-mean-square deviation (RMSD) of 0.7 Å. In particular, when we compared four *E*. *coli* GDH octamers, including the present GDH^NAD∙Gly^ structure and three other *E*. *coli* GDH structures (PDB: 5ZXL, 8GOA, and 8GOB), major structural differences were observed primarily at the β-hairpin and the region from helix ɑ2 to ɑ3 in the N-domain (Fig 4B), with an average RMSD of 2.8 Å for 62 C_ɑ_ atoms. These regions are where conformational changes occur depending on GDH’s ligand-bound state [17]. Nonetheless, the octameric interfaces were consistently maintained with an average RMSD of 0.5 Å. This feature was also observed when we compared *Bacillus stearothermophilus* GDH octamers from three different reported structures in distinct ligand-bound states (PDB: 1JPU, 1JQA, and 1JQ5), where the average RMSD was 0.3 Å for the interfaces (Fig 4C). Collectively, these findings show that octamerization of GDH is a consistent structural characteristic and independent of the ligand-binding state.

### Details in the octameric interfaces

**Fig 5:**
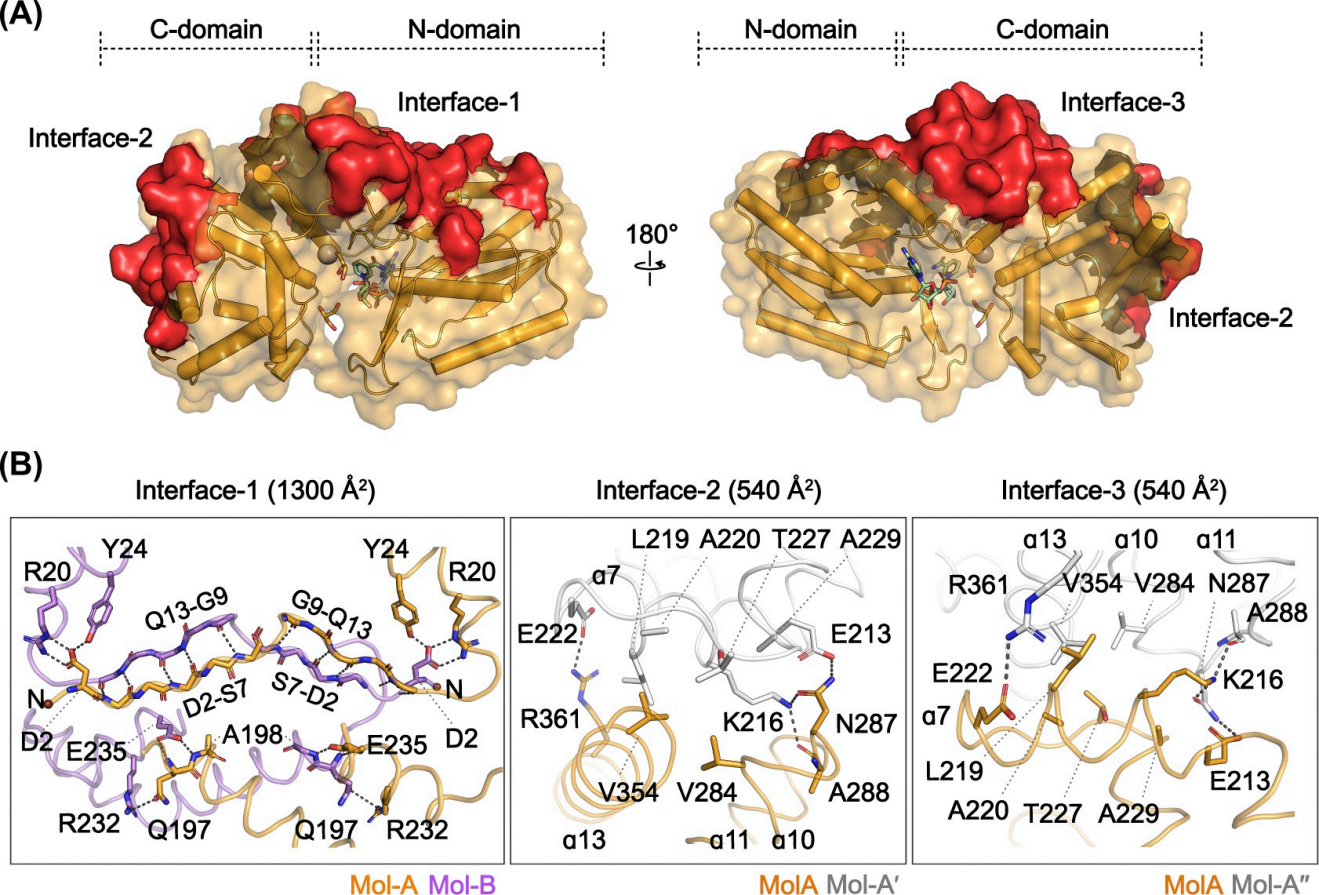
Details of the octameric interfaces in GDH^NAD∙Gly^. (A) Overall structure of Mol-A within the GDH^NAD∙Gly^ structure. The three octameric interfaces are shown as red surfaces. (B) Detailed views of the three octameric interfaces within the GDH^NAD∙Gly^ structure. Mol-A, Mol-B, and the neighboring protomers are colored orange, purple, and white, respectively. The side or main chains of the residues participating in the interactions are shown in stick with dashed lines indicating hydrogen bonds.

Among the three octameric interfaces, Interface-1 corresponds to the dimeric interface between Mol-A and Mol-B in the GDH^NAD∙Gly^ structure (Fig 5A). Within this interface, a region encompassing residues D2 to Q13 in one protomer interacts with the equivalent region in a neighboring protomer, forming two antiparallel β-sheets in a symmetrical fashion (Fig 5B). The backbones of residues R3, I5, and S7 on strand β1 in Mol-A respectively interact with the backbones of residues Q13, Y11, and G9 on strand β2 in Mol-B, and vice versa. Additionally, the side chain of D2 in Mol-A interacts with the side chains of R20 and Y24 in helix α1 in Mol-B, and nearby, Q197 at the N-terminus of helix α7 in Mol-A interacts with the side chains of R232 and E235 in helix α8 in Mol-B. These interactions also exhibit a symmetrical arrangement.

At Interface-2 of the GDH^NAD∙Gly^ octamer, a region including the α10–11 loop and helix α13 interacts with a region including the C-terminus of helix α7 and the α7–8 loop of a neighboring protomer (Mol-A′) (Fig 5B). The side chains or backbones of N287, A288, and R361 engage in electrostatic interactions at the edges of the interface with the side chains of E213, K216, and E222 in helix α7 of Mol-A′, while hydrophobic interactions occur at the center of the interface. These interactions were also seen at Interface-3, with corresponding residues reversed according to the 4-fold symmetry operation (Fig 5B). The region including the C-terminus of helix α7 and the α7–8 loop in Mol-A interacts with the region including the α10–11 loop and helix ɑ13 in another neighboring protomer (Mol-A″) through pairs equivalent to those at Interface-2.

**Fig 6:**
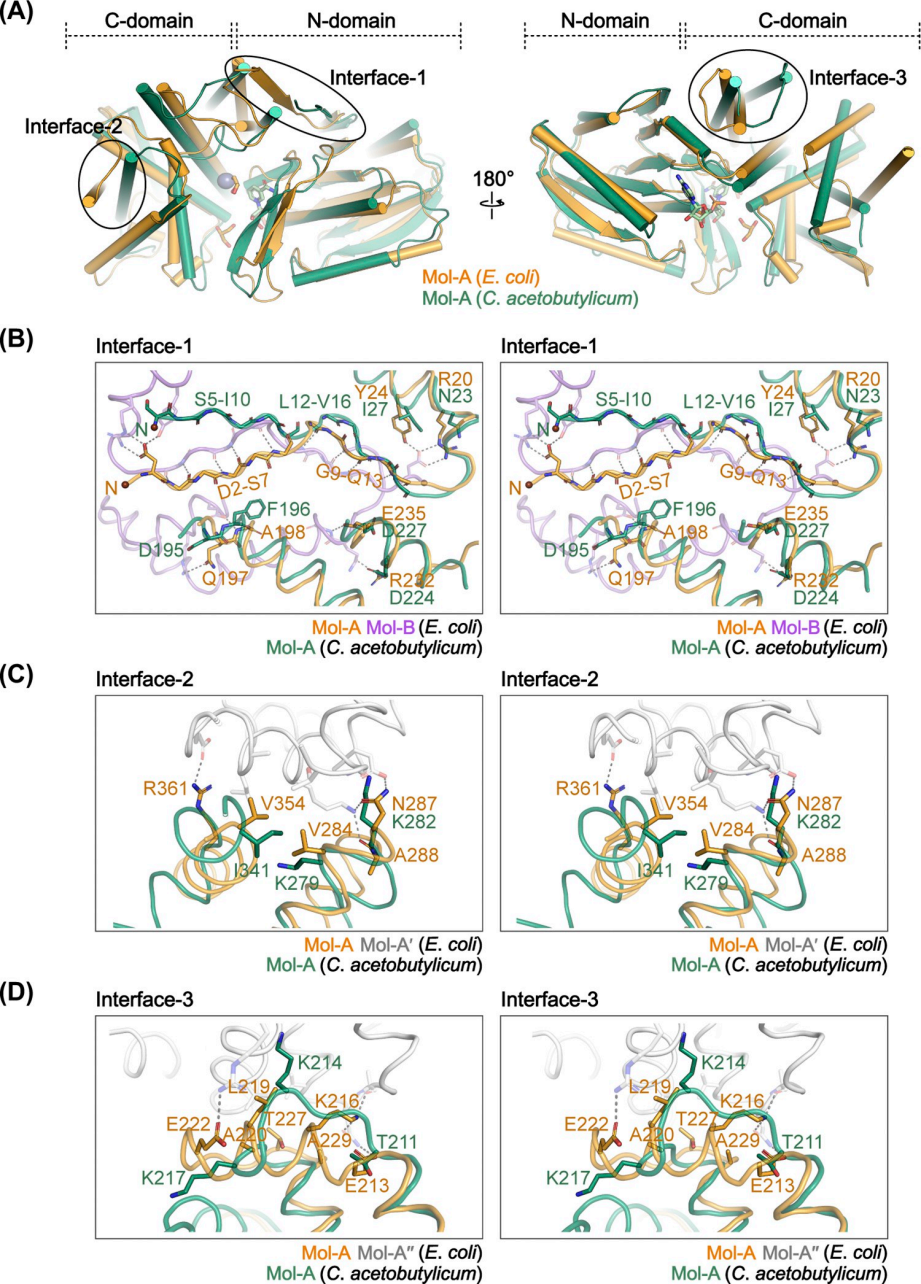
Structural comparison between *E*. *coli* and *C*. *acetobutylicum* GDHs. (A) Structural superposition of Mol-As of the GDH structures from *E*. *coli* (GDH^NAD∙Gly^) and *C*. *acetobutylicum* (PDB: 1TA9) colored orange and green, respectively. The three octameric interfaces of GDH are marked as black circles. (B-D) Stereo views (wall-eye) of the three octameric interfaces of the superimposed structure in (A). For *E*. *coli* GDH, neighboring protomers are presented together with transparency, and the residues participating in the interface interactions are shown in stick as in Fig 5B. The residues corresponding to the interface residues in *C*. *acetobutylicum* GDH and *E*. *coli* GDH are shown together.

By contrast, *C*. *acetobutylicum* GDH forms a completely distinct tetramer (Fig 3A). In *C*. *acetobutylicum* GDH, notable structural differences in the regions corresponding to the three interfaces (Fig 6A) mean that the interface interactions observed in all the other GDH structures were not maintained (Fig 6B–6D). Consequently, *C*. *acetobutylicum* GDH forms a totally different oligomeric arrangement, which illustrates the importance of the Interface-1, 2, and 3 for GDH octamerization.

### Octamerization reduces the structural dynamics of the GDH N-domain

In an earlier study, GDH exhibited notable structural dynamics in the β-hairpin region, which contributed to the enzyme’s function [23]. To further investigate the effect of octamerization on the structural dynamics of GDH, MD simulations were run using GDH octamer models. Given that the structural dynamics of the β-hairpin are influenced by the presence of a cofactor [23] and following the kinetic mechanism of GDH, where NAD^+^ binds first to the apo GDH [8–10], the octamer models used for simulation were prepared in the apo and NAD^+^-bound states. Additionally, an NAD^+^ and glycerol (NAD^+^∙glycerol)-bound state was included to investigate the potential impact of glycerol on the structural dynamics.

**Fig 7:**
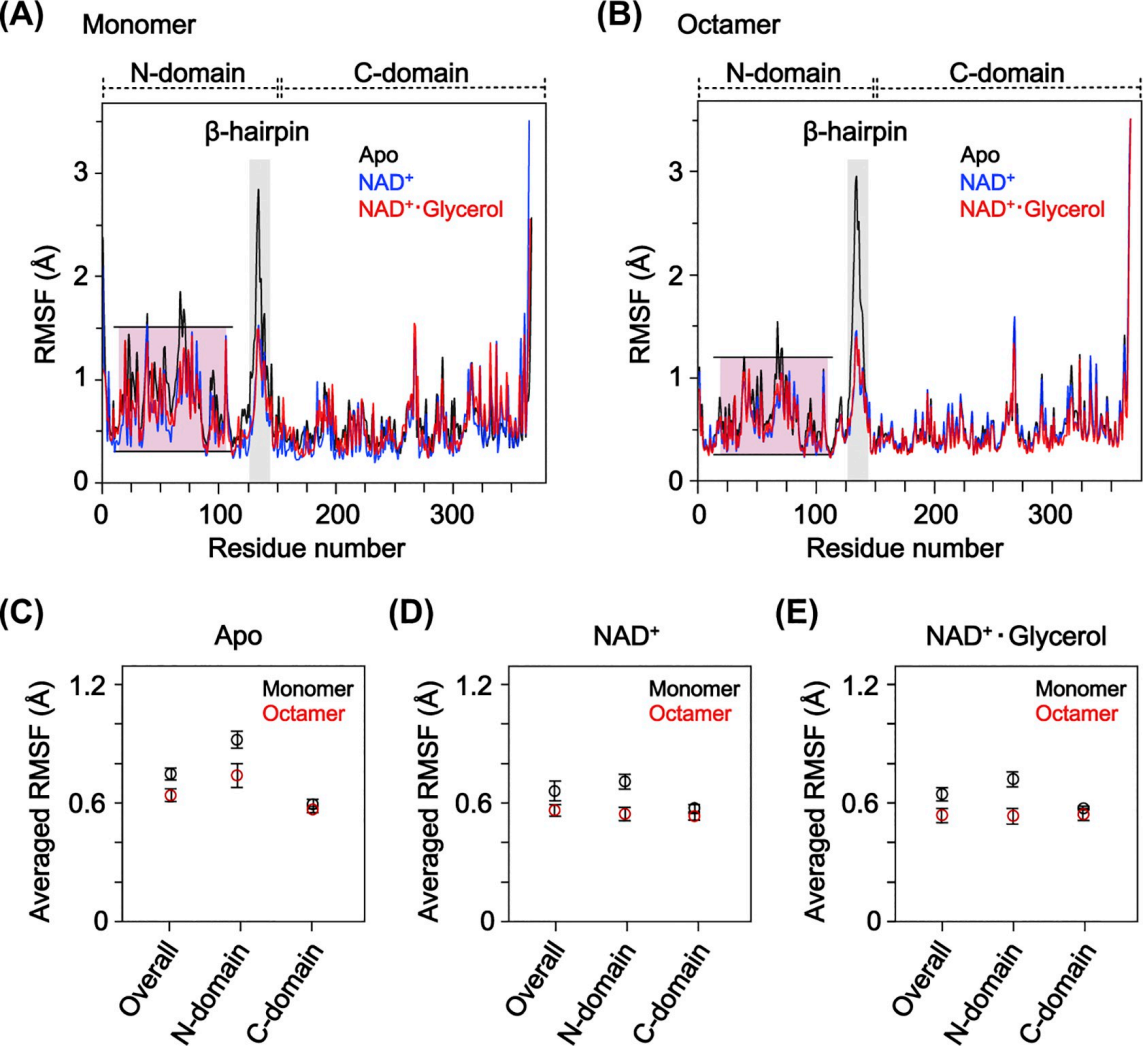
Comparison of the structural dynamics of the GDH monomer and octamer. (A-B) RMSFs per residue calculated from the MD simulations with the GDH monomer (A) and octamer (B) models in the apo (black), NAD^+^-bound (blue), and NAD^+^∙glycerol-bound (red) states. N-domain regions are marked with red boxes. (C-E) Averaged RMSFs for the model overall, the N-domain, and the C-domain from (A) and (B) in the apo (C), NAD^+^-bound (D), and NAD^+^∙glycerol-bound (E) states. Data are shown as the mean ± 95CI for triplicate experiments.

In the simulations, the dynamic properties of the β-hairpin were faithfully consistent with prior research [23], not only in the control monomer models but also in the octamer models (Fig 7A and 7B). Analysis of the RMSFs per residue, calculated from the simulation results, showed that within the octamer, the β-hairpin maintained the highest flexibility in the absence of NAD^+^. There was a noticeable decrease upon NAD^+^ binding, regardless of the presence of glycerol. These results demonstrate that octamer formation by GDH does not significantly alter the dynamic properties of the β-hairpin.

Intriguingly, however, for all three states the octamers exhibited an approximately 23% reduction in RMSFs within the N-domain as compared to the monomers (Fig 7C–7E). In each state, the RMSF in the C-domain showed negligible differences, with less than 5% variation between the monomer and octamer. By contrast, the RMSFs in the N-domain exhibited a 20% reduction in the octamers in the apo state, a 24% reduction in the NAD^+^-bound state, and a 25% reduction in the NAD^+^∙glycerol-bound state as compared to the monomers. These findings suggest that octamerization may contribute to stabilization of the GDH structure, particularly in the N-domain, by reducing its structural dynamics.

### Octamerization contributes to maintaining the distance between the ligands for catalysis

**Fig 8:**
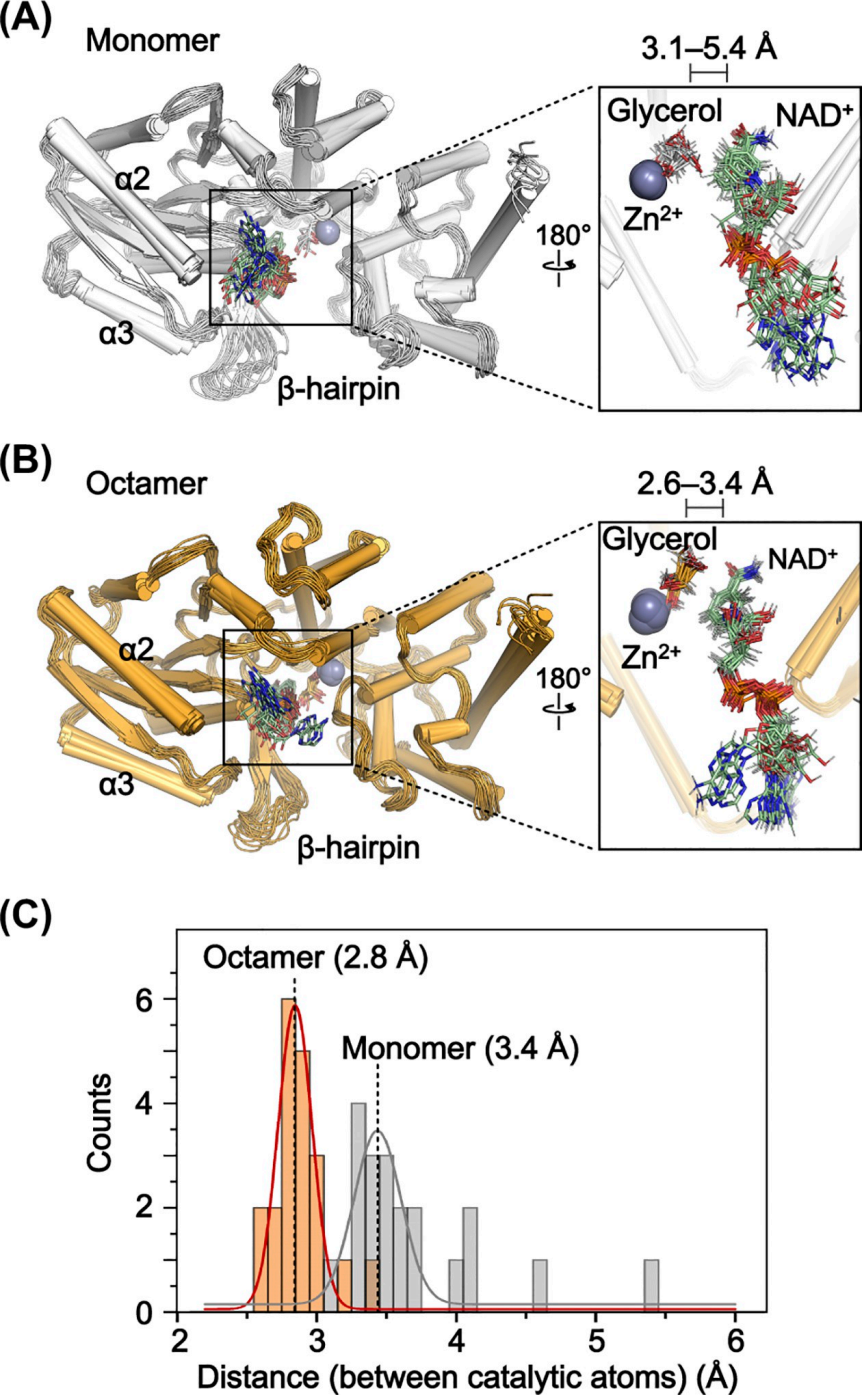
Comparison of distances between ligands in the GDH monomer and octamer. (A and B) Superposition of the 20 representative structures clustered from the results of the MD simulations with the GDH monomer (A) and octamer (B) models in the NAD^+^∙glycerol-bound state. NAD^+^ and glycerol are shown in stick, and Zn^2+^ is shown as a grey sphere. Detailed views of the active sites of each structure are presented with the distance ranges between the hydrogen atom of C_2_ of glycerol and the C_4_^N^ atom of NAD^+^. (C) Distances between the hydrogen atom of C_2_ of glycerol and the C_4_^N^ atom of NAD^+^ from (A) and (B) are plotted with curves calculated using Gaussian distribution fitting.

Given that the NAD^+^-binding residues within GDH are predominantly located in the N-domain, we hypothesized that the reduced dynamics of the N-domain may lead to more stable NAD^+^ binding and a more consistent distance between NAD^+^ and glycerol. To investigate this hypothesis, we conducted additional structural analyses with the simulation results. Initially, structural clustering was performed using the simulation result for the GDH monomer and octamer models in the NAD^+^∙glycerol-bound state. We selected 20 representative structures for each case, then superimposed them within each set, using Mol-A as a reference molecule for the octamer (Fig 8A and 8B). Subsequently, distances between the hydrogen atom of C_2_ of glycerol and the C_4_^N^ atom of NAD^+^, where the hydride shift occurs, were measured and compared.

Within the GDH monomer, the N-domain exhibits movements of up to 3.1 Å, leading to variation in the distance between the two atoms of up to 5.4 Å, with an average distance of 3.4 Å (Fig 8A and 8C). On the other hand, within the octamer, N-domain movements are restricted to a maximum of 2.0 Å, resulting in distances between the two atoms fluctuating up to 3.4 Å, with an average distance of 2.8 Å (Fig 8B and 8C). This distance is closer to the proposed distance between the two ligands where hydride shift occurs [11–13]. Within the octamer, the average distance was reduced by 18%, and the distribution was 27% narrower than was observed in the monomer (Fig 8C). These results indicate that within the octamer, the two atoms are consistently positioned closer together than in the monomer. Collectively, these findings demonstrate that octamerization of GDH reduces movement of its N-domain, allowing for a more consistent distance required for catalysis between the two ligands, potentially increasing the likelihood of the enzymatic reaction.

## Discussion

Earlier structural studies of GDH elucidated its active site and ligand binding [17, 19–23]. Studies also uncovered intriguing structural features, including a flexible β-hairpin for enzyme efficiency and the homo-octameric configuration of GDH [17, 21–23]. That research mainly focused on a binary complex of GDH and glycerol. Although the ternary complex with an inhibitor, Tris, was investigated, the complex with both the cofactor and true substrate had yet to be investigated. In addition, the structural details of the octamerization, which is a noteworthy structural feature of GDH, and its potential impact on the enzyme’s activity remained unexplored.

In the present study, we report the structure of the ternary complex of GDH with both NAD^+^ and glycerol bound. Comparison of the GDH^NAD∙Gly^ ternary structure and the previously reported ternary structure with Tris (PDB: 8GOB) revealed their high degree of similarity, with an RMSD of 0.3 Å. Additionally, they showed similarity to another NAD^+^-bound GDH structure where the substrate was absent (PDB: 1JQ5; RMSD of ~0.6 Å), despite a sequence identity of only 49%. Our earlier study revealed that GDH exhibits structural changes dependent on the presence of NAD^+^ at the active site [23]. Consistent with that finding, the aforementioned three NAD^+^-bound GDH structures differed from structures lacking NAD^+^, with RMSDs of 1.0–2.6 Å. These comparisons not only support the earlier finding but also suggest that GDH maintains a consistent structure once NAD^+^ is bound at the active site, regardless of the presence or type of substrate.

Within the GDH^NAD∙Gly^ structure, the two non-crystallographic protomers exhibited distinct ligand binding. In Mol-A, in contrast to the active site NAD^+^, two glycerol molecules were found adopting improper positions for catalysis. One glycerol was found at the active site, coordinated with Zn^2+^ in a non-productive manner such that the C_2_-OH oxygen was not involved in the coordination. In other reported GDH structures, the active site glycerol also showed either a dual conformation with both productive and non-productive orientations (PDB: 1JQA) or another non-productive orientation where both the C_1_-OH and C_3_-OH oxygens participated in the Zn^2+^ coordination (PDB: 4MCA). This suggests that the active site glycerol does not consistently maintain a specific binding pose under different conditions and is relatively dynamic, possibly due to the absence of specific residues that directly bind glycerol.

The other glycerol in Mol-A was bound to the secondary binding site, as observed in several GDH structures (PDB: 3UHJ, 5XN8, 8GOA, and 8GOB). However, the structural and functional significance of this site remains unexamined. Given its exposure to the solvent and the presence of polar or positively charged residues, such as His257 and Lys274 (Fig 2B), we suggest that it may facilitate the capture of GDH’s polyol substrate from the solvent. Additionally, the proximity of oxygen atoms in the substrates bound to this site to the O_2_^N^ atom of NAD^+^ (3.2–4.0 Å) suggests a potential role in stabilizing the prior bound NAD^+^ [23]. Alternatively, it may just represent a trace of the substrate entry path, as hypothesized in an earlier report [19].

By contrast, glycerol did not bind in Mol-B. Furthermore, NAD^+^ was bound at an alternative site along the central cleft (Figs 1 and 2), possibly in an intermediate state entering or leaving the active site. Although the fitting of NAD^+^ to Mol-B was reasonable considering the shape of the electron density map and the crystallization conditions, its electron density was a less defined molecular feature than in Mol-A (S2 Fig). In addition, the atomic displacement parameter, B-factor, for NAD^+^ was higher in Mol-B (Table 1), indicating that Mol-B’s NAD^+^ is less ordered than in Mol-A, where it is bound at the active site. To minimize misfitting, we carefully fitted the NAD^+^ molecule based on the calculated simulated annealing omit *F*_o_-*F*_c_ maps and the surrounding residues.

Despite the distinct ligand binding observed among the protomers, our structure also exhibited octameric formation, consistent with the other reported GDH structures. Through structural comparison, we revealed that the octamerization of GDH is maintained by three interfaces, which, if not formed, result in a completely different oligomeric arrangement (Figs 5 and 6), and that this octamerization is a consistent structural feature of the enzyme, irrespective of its ligand-bound state (Figs 3 and 4). Together, the three octameric interfaces form a total interface area of approximately 2380 Å^2^ per protomer across the two domains. Nevertheless, it is worth noting that the active site and the N-domain regions, which undergo structural changes upon ligand binding, are not directly involved in these interfaces. Accordingly, each protomer of the GDH octamer could independently bind ligands, as was observed in the GDH^NAD∙Gly^ structure.

Moreover, we found that the octamerization of GDH reduces the structural dynamics of the N-domain. This decreases the dynamics of the bound NAD^+^ and promotes a more consistent distance between the ligands (Figs 7 and 8). The simulation results further indicate that both NAD^+^ and glycerol exhibit decreased dynamics within the octamer, suggesting the reduced dynamics of NAD^+^ may further reduce glycerol’s dynamics, which would contribute to maintaining a consistent distance between the ligands. These findings collectively suggest that GDH’s octamerization enhances the likelihood of the catalytic reaction. In addition, the less dynamic nature of the N-domain within the octamer appears to enhance ligand binding by promoting a more stable and rigid configuration, as described above. However, this reduction in dynamics could introduce more spatial constraints within the octamer during the ligand entry and exit processes, as compared to the monomer. On the other hand, the β-hairpin’s dynamics in apo GDH, which contribute to ligand binding and, potentially, ligand entry and exit [23], are consistently maintained irrespective of the oligomeric state (Fig 7A and 7B). It therefore seems unlikely that the less dynamic N-domain within the octamer significantly interferes with ligand entry and exit. That said, further investigation will be needed to resolve this issue.

The octamerization may also expedite the enzymatic reaction of GDH through a concentration effect. An enzyme’s reaction rate tends to increase as its concentration rises due to increases in the proportion of enzyme-substrate complex. Oligomerization of an enzyme, as in the case of GDH, could induce this effect by leading to a higher local concentration of the enzyme in a substrate’s vicinity as compared to when only the monomeric form is present. In addition, oligomerization generally enhances a protein’s thermal stability; indeed, it is known to be one of the factors responsible for the thermal stability of thermophilic proteins [43–46]. Octamerization of GDH is therefore also expected to contribute to its thermal stability, as indicated by its high *T*_m_ [14–16].

In this report, we highlighted octamerization as a noteworthy structural feature of GDH and proposed its potential contribution to the enzyme activity, acknowledging that further experiments will be necessary to validate this suggestion. Our structural analysis enhances our comprehension of the GDH structure and its relation to its enzymatic activity.

## Supporting information

S1 Fig Size-exclusion chromatogram. (A) Size-exclusion chromatogram of *E*. *coli* GDH full-length (blue line) compared to standards (black solid or dashed lines). The inset contains SDS-PAGE results for the fractions corresponding to GDH. (B) Standard curve generated from a linear fit of the log(molecular weight(kDa)) of the standards versus their elution parameter *K*_av_. The molecular weight of GDH was estimated to be 330 kDa (≈40 kDa for monomer × 8) based on the standard curve.
(TIF)

S2 Fig NAD^+^ with its simulated annealing omit *F*_o_-*F*_c_ map. (A and B) NAD^+^ in Mol-A (A) and Mol-B with its simulated annealing omit *F*_o_-*F*_c_ maps (green meshes) contoured at 3.0 σ.
(TIF)
