# Supplementary figures and images for: Structural insights into the octamerization of glycerol dehydrogenase
Source: PLoS One. 2024 Mar 14;19(3):e0300541. doi: 10.1371/journal.pone.0300541 (PMC10939272; doi:10.1371/journal.pone.0300541)

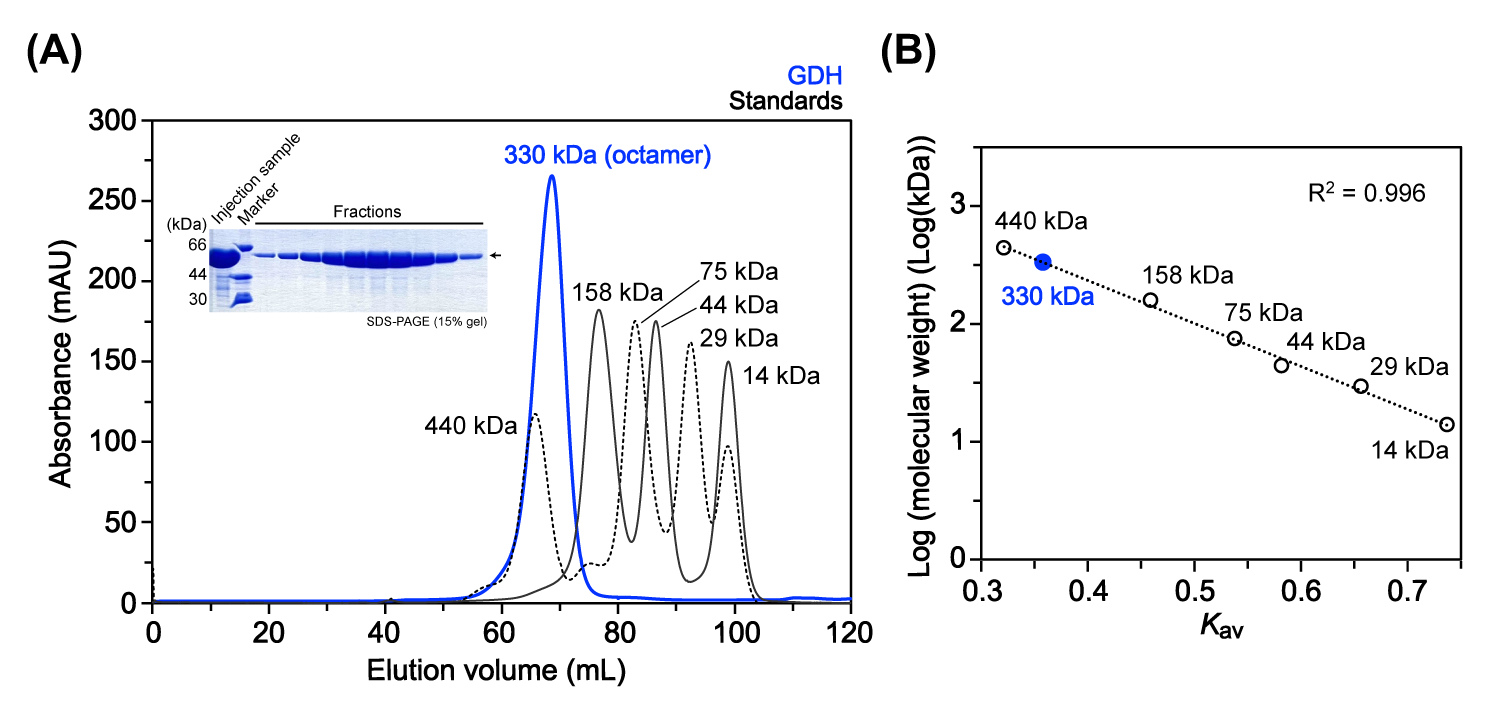

Supplement: S1 Fig — (A) Size-exclusion chromatogram of E. coli GDH full-length (blue line) compared to standards (black solid or dashed lines). The inset contains SDS-PAGE results for the fractions corresponding to GDH. (B) Standard curve generated from a linear fit of the log(molecular weight(kDa)) of the standards versus their elution parameter Kav. The molecular weight of GDH was estimated to be 330 kDa (≈40 kDa for monomer × 8) based on the standard curve. (TIF) [file pone.0300541.s001.tif]

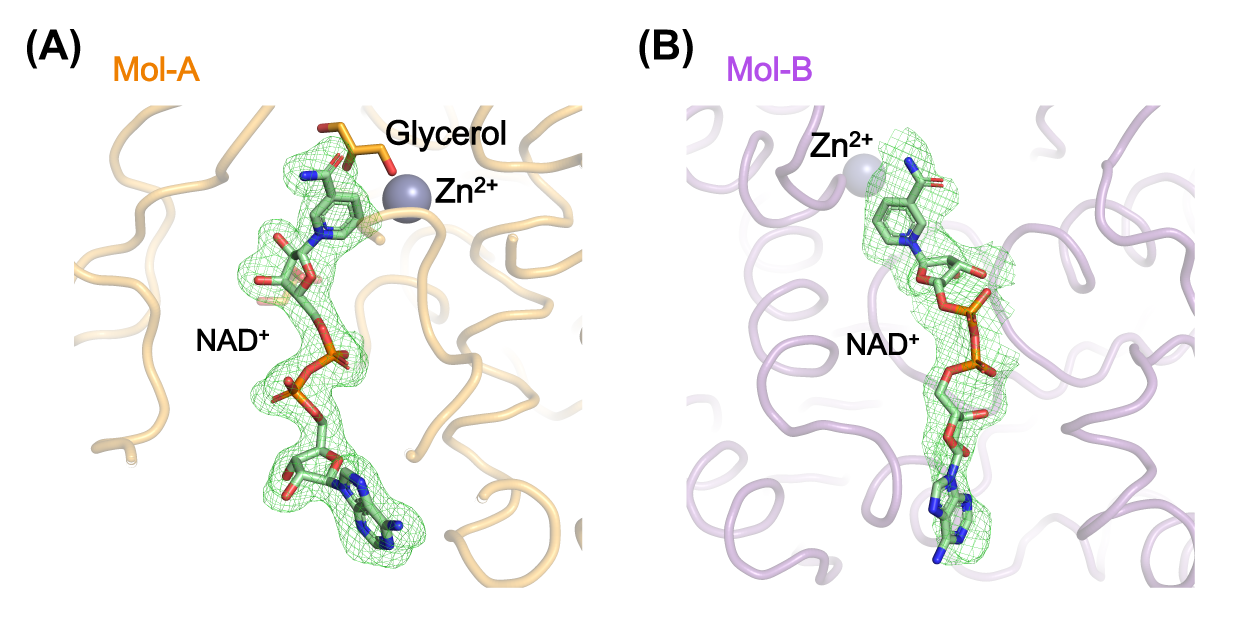

Supplement: S2 Fig — (A and B) NAD+ in Mol-A (A) and Mol-B with its simulated annealing omit Fo-Fc maps (green meshes) contoured at 3.0 σ. (TIF) [file pone.0300541.s002.tif]
